# Au nanobipyramids with Pt decoration enveloped in TiO_2_ nanoboxes for photocatalytic reactions

**DOI:** 10.1039/d1na00092f

**Published:** 2021-06-01

**Authors:** Weijian Gao, Caixia Kan, Shanlin Ke, Qinru Yun, Xingzhong Zhu, Xiaoguang Zhu

**Affiliations:** College of Science, Nanjing University of Aeronautics and Astronautics Nanjing 210016 China cxkan@nuaa.edu.cn; Key Laboratory of Aerospace Information Materials and Physics, Ministry of Industry and Information Technology Nanjing 210016 China; Institute of Solid State Physics, Chinese Academy of Sciences Hefei 230031 China; Key Laboratory for Intelligent Nano Materials and Devices of the Ministry of Education Nanjing 210016 China

## Abstract

Noble metal nanocrystals and core–shell nanocomposites have attracted particular interest due to their unique optical properties originating from surface plasmon resonance (SPR) and wide applications related to the SPR effect. In this work, we designed and fabricated a new Au–Pt@TiO_2_ nanocomposite, in which Au nanobipyramids (AuNBPs) decorated with platinum (Pt) clusters were enveloped in mesoporous TiO_2_ nanoboxes with nanocavities. AuNBPs provide strong SPR absorption and localized field enhancement restricted to the cavities of TiO_2_ nanoboxes. The Pt nanoclusters decorated on the surface of AuNBPs can effectively modulate the charge movement and energy transfer in the photocatalytic process. The enhanced electric field provides a local thermal effect for the photocatalytic reaction and promotes the injection process of hot electrons which facilitates carrier separation. The nanoboxes with nanocavities can effectively manage the usage of localized energy and provide space for reaction. Under the cooperative effects, the photocatalytic performance was remarkably improved along with durability and stability. For the AuNBP–Pt@TiO_2_ nanoboxes, the rhodamine-B degradation efficiency was ∼6.5 times that of AuNBP@TiO_2_ nanoboxes. The mechanism of the photocatalysis process was proposed based on experimental results and simulations. Benefiting from the excellent structure and properties, the obtained nanostructure is a promising candidate in the fields of pollutant degradation and chemical reaction catalysis.

## Abstract 2

In this work, we designed and fabricated a new Au–Pt@TiO_2_ nanocomposite. Lead to the effective synergy between components, it show remarkable photocatalytic activity.

## Graphical abstract

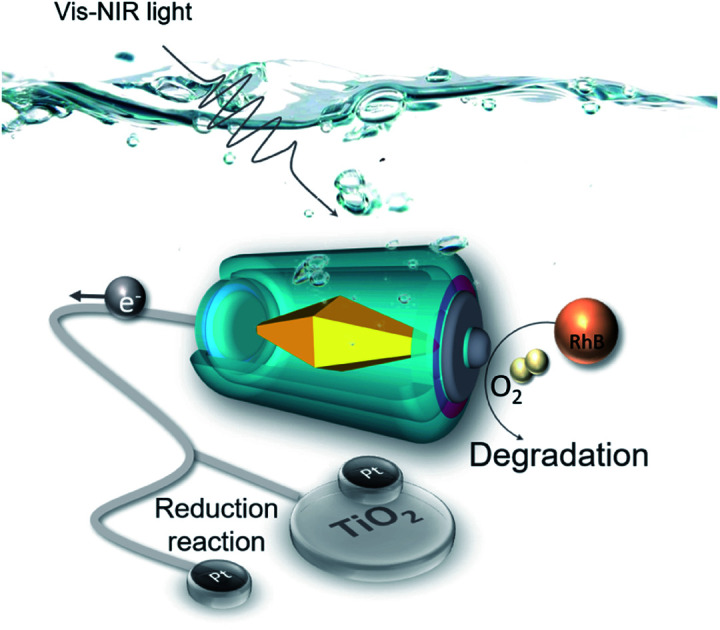

## Introduction

Photocatalysis is a catalytic reaction that directly converts solar energy into chemical energy. In photocatalysis technology, it is important to design and fabricate a stable structure with recyclability and high energy utilization. Up to now, researchers have studied the synthesis and catalytic performance of a variety of substances and structures.^1–4^ Usually, much attention is paid to the photoelectric efficiency and catalytic performance of metal oxide photocatalysts.^5,6^ Most experiments were carried out in an open environment, which inevitably led to a loss of large amounts of energy. Therefore, it is worth studying how to effectively manage the light energy in photocatalytic reactions. On account of the unique surface plasmon resonance (SPR) effect and the tunable resonance absorption of incident light in a wide spectral region, noble metal nanostructures can effectively solve the above problems.^7,8^ The highly localized electric field and the hot electron effect of noble metal nanostructures play effective roles in promoting charge separation in photocatalytic reactions. According to previous reports,^9–12^ in metal@semiconductor nanocomposites, the SPR effect of metal nanostructures can improve the plasmon-induced resonance energy transfer (PIRET)^13,14^ and hot electron injection (HEI),^15–17^ which enhance the light utilization range and energy conversion. Particularly, for wide-gap semiconductors with excellent photocatalytic properties and little absorption of visible and near-infrared (vis-NIR) light, extending the light utilization range to vis-NIR light was a significant aspect in photocatalyst design.^18,19^ The PIRET relies on the spectral overlap between semiconductors and noble metal nanostructures.^20^ In contrast, the HEI process can transfer high-energy hot electrons from metal nanostructures to the conduction band of adjacent semiconductors without spectral overlap with the semiconductors.^16^ Thus, the HEI effect was considered as an excellent strategy to design photocatalysts with catalytic activity under vis-NIR light. For the HEI process, resonant plasmons localized to metal nanostructures are attenuated in a non-radiative attenuation process *via* interband excitation and converted to electron–hole pairs with energy higher than the metal’s Fermi energy.^21^ Then, the hot electrons are injected into the adjacent semiconductor conduction band, leading to an increase of the carrier concentration and electron migration rate.^7,22^ As a result, the catalytic reaction would be greatly promoted. The HEI process has been extensively studied in improving photocatalysis in recent years.^23^ For example, Au/TiO_2_ nanocomposites have been applied in hydrogen production from water,^24,25^ photooxidation of organic molecules,^24^ and the CO_2_ reduction reaction^26,27^ by the HEI process. Among the nanostructures with an intense SPR effect, gold nanorods (AuNRs) have been widely studied in the past two decades for their unique optical properties and potential applications.^28,29^ Compared with Au nanorods, Au nanobipyramids (AuNBPs) show greater local electric field enhancement, larger optical cross-section, narrower line widths, and better size uniformity.^30^ AuNBPs were synthesized later than AuNRs, and publication of related reports began in 2007.^31^ Then in 2015, an AuNBP purification strategy proposed that the purity of AuNBPs can reach close to 100%.^32^ Subsequent publications were focused on the fabrication of complex nanostructures and their application based on the as-obtained AuNBPs.^33–35^ More recently, Kan *et al*.^36^ achieved strong SERS signal enhancement by coating an alloy shell of Au and Ag on AuNBPs. AuNBP-embedded silver–platinum hollow nanostructures^37^ were fabricated to monitor stepwise reduction and oxidation reactions. Their excellent electric field enhancement effect strongly promotes the generation of hot electrons and holes. The large extinction cross-section can enhance the photocatalytic performance over a wide controllable spectral range through plasmonic sensitization. The design of AuNBP-based nanocomposites has led to the optimization of the photocatalytic activity.^38^ Wang *et al*.^35^ combined good SERS performance with catalytic activity by embedding AuNBPs into a nanoframe composed of a catalytic metal. In the AuNBP/semiconductor composite system, charge carriers' separation is largely promoted by the strong electric field enhancement of AuNBPs. For instance, the AuNBP@Cu_2_O nanostructure fabricated by Yang *et al*.^39^ shows 20 times enhanced photocatalytic efficiency in the degradation of methyl orange under visible light compared to pure Cu_2_O nanoparticles. Irina Levchuk *et al*.^40^ enhanced photocatalytic activity through insertion of AuNBPs into porous TiO_2_/SiO_2_ hybrid composite films. It is reported that TiO_2_-coated AuNBPs can be used for blocking autophagy flux and sensitizing cancer cells to proteasome inhibitor-induced death.^41^ In addition to the direct use of AuNBPs, modifying with co-catalysts is an efficient approach to upgrade photocatalytic activity. Wang *et al*.^42^ achieved several times enhanced photocatalytic activity in hydrogen production, which was attained by surface modification with Pt nanoparticles in Au–TiO_2_ nanodumbbells. Consequently, reasonable design of AuNBP-based photocatalysts is critical for their excellent properties in photocatalytic reactions.^43^ In this work, we designed and fabricated a new AuNBP–Pt@TiO_2_ nanostructure, in which AuNBPs decorated with Pt clusters were enveloped in mesoporous TiO_2_ nanoboxes with nanocavities. By subsequent growth of a Ag shell and a mesoporous TiO_2_ outer shell on the surface of AuNBPs, the cavities were constructed after removal of the Ag shell, which provides a superior reaction space for the photocatalytic reaction and promotes better separation of carriers. Simultaneously, Pt nanoclusters increase the photocatalytic efficiency of the organic degradation reaction. Finite-difference-time-domain simulation was performed to verify the enhancement and electric field distribution of the nanostructures. The active synergy between the semiconductor nanoboxes and AuNBPs expands the respective applications of the components and provides an effective strategy for the establishment of the photocatalysis process.

## Experimental

### Materials

Hydrogen tetrachloroaurate trihydrate (HAuCl_4_·3H_2_O, 99%), chloroplatinic acid hexahydrate (H_2_PtCl_6_, 99%), sodium borohydride (NaBH_4_, 98%), trisodium citrate (TSC, 99%), ascorbic acid (AA, 99%), iron nitrate nonahydrate (Fe(NO_3_)_3_·9H_2_O, 99%) and silver nitrate (AgNO_3_, 99%) were purchased from Sigma-Aldrich. Cetyltrimethylammonium bromide (CTAB, 98%) was obtained from Alfa Aesar. Hydrogen peroxide solution (H_2_O_2_, 30 wt% in water), cetyltrimethylammonium chloride (CTAC, 97%), sodium hydroxide (NaOH, 96%), ammonia solution (NH_3_·H_2_O, 25 wt% in water), titanium chloride solution (TiCl_3_, 15–20% in 30% HCl), sodium bicarbonate (NaHCO_3_, 99%), sodium dodecyl sulfate (SDS, 98%), nitric acid (HNO_3_, 67% in water), hydrochloric acid solution (HCl, 37 wt% in water), rhodamine B (RhB, 99%) and salicylic acid (SA, 99%) were purchased from Aladdin Reagent. All chemicals were used without further purification. Deionized water with a resistivity of 18.2 MΩ cm produced from a Direct-Q5 ultraviolet water purification system was used in all experiments.

### Synthesis of AuNBPs

As reported previously,^8,31,32,36^ a seed-mediated method was used to fabricate AuNBPs. A fresh ice-cold NaBH_4_ solution (0.15 mL, 0.01 M) was injected quickly into a 10 mL aqueous solution of HAuCl_4_ (0.125 mL, 0.01 M) and TSC (0.25 mL, 0.01 M). The resultant seed solution was kept at 85 °C for 1.5 h to promote the formation of five-fold twined Au seeds. The growth solution was made by sequential addition of HAuCl_4_ (0.05 M, 1 mL), AgNO_3_ (0.02 M, 0.5 mL), HCl (1 M, 2 mL), and AA (0.1 M, 0.8 mL) to the CTAB solution (0.1 M, 100 mL), and then shaking gently until the solution becomes colorless. Then the as-prepared seed solution (0.26 mL) was injected into the growth solution under gentle stirring for 10 s. The reaction solution was kept overnight at room temperature for the growth of AuNBPs. Typically, AuNBPs in high yield are achieved by two steps, namely Ag overgrowth based on the as-grown AuNBPs and the undisturbed storage and re-etching with NH_3_·H_2_O (8 mL) and H_2_O_2_ (6%, 5 mL) solution.

### Synthesis of AuNBP@TiO_2_ nanoboxes

The AuNBP@TiO_2_ nanoboxes were prepared in three steps. First, Au@AgNRs were prepared by a chemical reduction method.^35,43^ The AuNBPs were redispersed in a CTAC solution (10 mL, 0.08 M), followed by the subsequent addition and mixing of AgNO_3_ (150 μL, 0.02 M) and AA (150 μL, 0.1 M) and then stirred for 4.5 h to grow Ag shells on the AuNBPs in the form of nanorods (Au@AgNRs). The Au@AgNRs were washed with deionized water and redispersed in an SDS solution (20 mL, 0.1 M). The cationic surfactant-encapsulated Au@AgNRs were exchanged with the SDS anionic surfactant. After 30 min and the removal of excessive SDS, the Au@AgNRs were redispersed in 1 mL of deionized water for further mesoporous TiO_2_ shell coating. For the surface TiO_2_ coating, 50 μL of 15–20% TiCl_3_ solution was diluted with 4 mL of water followed by dropwise injection of NaHCO_3_ solution (750 μL, 0.6 M). Then the as-prepared Au@AgNR colloid was added to the system. (In the synthesis of pure amorphous TiO_2_, the as-prepared Au@AgNR colloid was replaced by SDS (0.2 mL, 0.1 M), followed by gentle stirring for 2 h.) The dispersion was kept under gentle stirring for TiO_2_ coating on the surface of Au@AgNRs. The products after reaction for 30 min (Au@AgNR@TiO_2_) were collected by centrifugation and washed with ethanol and water (65 °C) for removing the residual chemicals. The products were redispersed in 20 mL of deionized water. For the AuNBP@TiO_2_ nanobox formation, the Ag shells on the AuNBP surface were removed by adding 200 μL of HNO_3_ (0.1 M) and 100 μL of Fe(NO_3_)_3_ (0.02 M). The mixture was stirred for 1 h. Through this process, nanoboxes with cavities between the AuNBP cores and TiO_2_ mesoporous shells were formed. The sample was washed with ethanol and stored in 20 mL of water for further use.

### Synthesis of AuNBP–Pt@TiO_2_ nanoboxes

AuNBP–Pt@TiO_2_ nanoboxes were prepared by a chemical reduction method.^42^ 20 mL of the as-prepared AuNBP@TiO_2_ nanoboxes was mixed with 200 μL of 0.1 M AA after adding 30 μL of 1 mM aqueous H_2_PtCl_6_ solution. The mixture was maintained at 65 °C for 8 h for the formation of Pt clusters (Pt clusters were more easy to grow on the surface of AuNBPs). Then the sample was centrifuged and re-dispersed in deionized water.

### Photocatalytic measurements

Photocatalytic organic degradation tests were performed in a 50 mL quartz reactor. 1 mL of the as-synthesized samples was diluted to 20 mL with an aqueous solution making the final concentration of RhB 0.3 mM (0.25 mM SA) and sonicated for 10 min. A Xe-lamp (CEL-HXF300) was employed as the irradiation source with light filtered using a GG-420 filter(>420 nm). Magnetic stirring was applied to keep the photocatalyst particles suspended in the solution throughout the experiment. Organic degradation was measured periodically with a UV-vis spectrometer. 400 μL of reaction mixture was collected per 10 min and centrifuged at 10 000 rpm for 10 min to remove the solid catalyst. Further, a cycling stability experiment was performed under the same conditions.

### Finite-difference-time-domain simulation

FDTD simulations were performed using FDTD Solutions 8.7 (Lumerical Solutions) to simulate the bare AuNBPs, AuNBP@TiO_2_ core–shell structures, and AuNBP@TiO_2_ nanoboxes. During the simulations, an electromagnetic pulse in the spectral range from 200 nm to 1100 nm was launched into a box containing a target nanostructure. The refractive index of the surrounding medium was 1.33, the same as that of water. The dielectric function of Au was obtained by fitting the measured data of Johnson and Christy,^44^ and Pt was fitted from Palik's data.^45^ The sizes of the AuNBPs and the AuNBP-based nanostructures were set according to the average waist widths and lengths measured from the TEM images. The refractive index of the amorphous TiO_2_ shell was set as 1.65.^46^ The boundary with different nanostructures inside was divided into meshes of 1 nm size.

### Characterization

The morphology of the as-prepared samples was characterized by transmission electron microscopy (TEM: JEOL-100CX, Japan) and energy-dispersive X-ray spectroscopy (EDS: TecnaiG2 F20). Ultraviolet-visible (UV-vis) absorption spectra were recorded with a UV-vis spectrometer (3600-Plus spectrophotometer). X-ray diffraction (XRD) spectra were recorded with an X-ray diffractometer (Panalytical Empyrean).

## Results and discussion

**Fig. 1:**
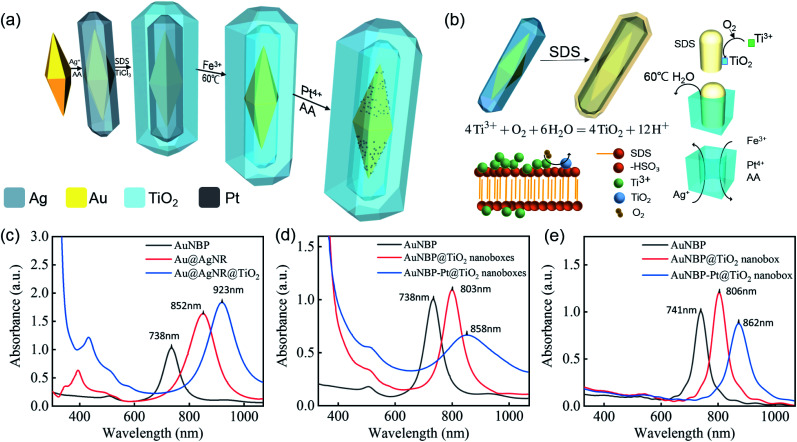
(a) The diagram of the synthesis process of the AuNBP–Pt@TiO_2_ nanoboxes, (b) the function of the SDS soft template in the formation of the mesoporous structure, (c and d) optical absorption spectra of samples in the synthesis process, (e) FDTD-simulation optical spectra of AuNBPs, AuNBP@TiO_2_ nanoboxes and AuNBP–Pt@TiO_2_ nanoboxes.

The graphic illustration of the synthesis process of AuNBP–Pt@TiO_2_ nanoboxes is shown in Fig. 1a. The formation process of the TiO_2_ shell and the role played by SDS are illustrated in detail in Fig. 1b. SDS micelles segregate to the Au@AgNR surface. The hydrophilic end of SDS is negatively charged in water, which would attract the titanium tri-positive ions (Ti^3+^). As the pH reaches the hydrolysis limit of Ti^3+^, oxygen oxidized Ti^3+^ to TiO_2_ which was deposited on the surface of Au@AgNRs to form an outer shell.^47^ Since the solubility of SDS increases sharply, after centrifugal washing with 65 °C deionized water, the SDS molecules were dissolved from the shell and removed by the water, leading to the formation of a mesoporous TiO_2_ shell.

The optical spectra of nanostructures sampled in the preparation process are shown in Fig. 1c and d. The AuNBP@TiO_2_ nanoboxes present a strong absorption band at about 803 nm and a weaker peak at about 505 nm (Fig. 1d), which are ascribed to the longitudinal SPR (SPR_L_) and transverse SPR (SPR_T_) of AuNBPs,^48^ respectively. Due to the formation of TiO_2_ nanoboxes, the refractive index around AuNBPs was changed. Different from the core–shell nanostructure, only the transverse part of AuNBPs was in direct contact with the TiO_2_ nanoboxes. Because of the higher polarizability of the AuNBPs at the SPR_L_ than at the transverse one, the SPR_L_ peak has higher sensitivity. Therefore, the SPR_L_ red shifts from 738 nm to 803 nm with a full width at half maximum (FWHM) of ∼70 nm. The strong absorption of wavelength below 400 nm was produced by the typical bandgap transition of TiO_2_ (∼3.2 eV). After the modification with Pt clusters, the peak of SPR_L_ red shifted to ∼860 nm with a broad FWHM of ∼210 nm. The changes of absorption spectra were mainly caused by the random growth of Pt clusters on the surface of AuNBPs. Corresponding to the graphic illustration and the TEM images, FDTD solutions were applied to simulate the optical spectra of AuNBPs, AuNBP@TiO_2_ nanoboxes and AuNBP–Pt@TiO_2_ nanoboxes, which are shown in Fig. 1e. The FDTD method is regarded as a powerful and flexible technique for studying the optical properties of metal nanostructures.^36^ The plasmon absorption peaks of the AuNBPs, AuNBP@TiO_2_ nanoboxes and AuNBP–Pt@TiO_2_ nanoboxes were calculated to be at ∼741 nm, ∼806 nm, and ∼862 nm, respectively, which were in accordance with previous data obtained from experimental measurements. The results indicated that the following FDTD electric field simulations results were reliable.

**Fig. 2:**
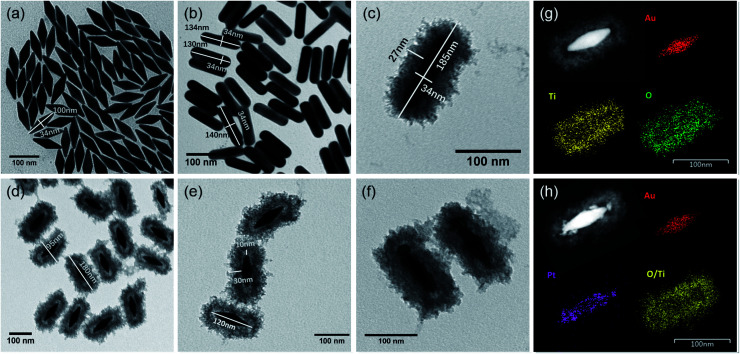
The TEM images of (a) AuNBPs, (b) Au@AgNRs, (c) Au@AgNR@TiO_2_, (d and e) AuNBP@TiO_2_ nanoboxes, and (f) AuNBP–Pt@TiO_2_ nanoboxes. (g and h) HAADF-STEM image and elemental maps of AuNBP@TiO_2_ nanoboxes and AuNBP–Pt@TiO_2_ nanoboxes.

**Fig. 3:**
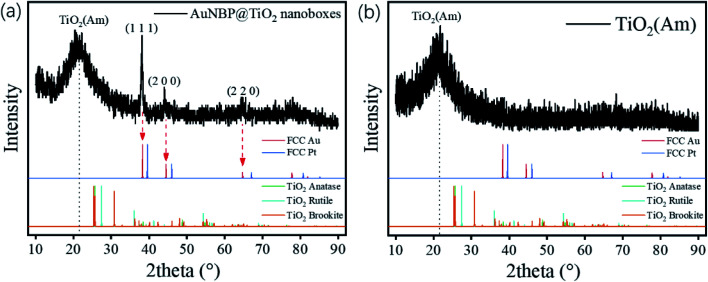
XRD patterns of the (a) AuNBP@TiO_2_ nanoboxes and (b) amorphous TiO_2_.

The designed morphologies were confirmed using TEM images (see Fig. 2a–f). AuNBPs were 100 nm ± 2 nm in length and 34 nm in diameter. After being coated with Ag nanoshells in the form of nanorods, the diameter of AuNBPs shows no obvious increase, and the two sharp ends of AuNBPs become round. It can be seen from Fig. 2c that the thickness of the TiO_2_ shell is ∼30 nm. Owing to the mesoporous shell of the structure, Ag could be easily removed from the inside of the TiO_2_ shell, which led to the formation of nanoboxes with cavities (Fig. 2d and e). After the removal of SDS and the Ag shell, AuNBPs were mainly located in the middle of the TiO_2_ nanoboxes which indicated the direct connection between the middle of AuNBPs and the TiO_2_ nanoboxes. The morphology of the mesoporous TiO_2_ nanoboxes showed no obvious changes after the removal of the Ag layer by Fe^3+^, which specified the structural stability of the mesoporous shell. By comparing the element distribution of AuNBP@TiO_2_ nanoboxes and AuNBP–Pt@TiO_2_ nanoboxes through the element distribution mapping (Fig. 2g and h) obtained from energy dispersive spectroscopy, it can be seen that most Pt clusters are grown on the surface of AuNBPs, which confirms the porous model of the TiO_2_ shell. Usually, XRD is applied to characterize the crystalline phases of prepared materials. The XRD results for the AuNBP@TiO_2_ nanoboxes and the prepared amorphous TiO_2_ powder are shown in Fig. 3a and b. Because the porous TiO_2_ was prepared at room temperature without a further high temperature annealing process, the characteristic peaks of common crystalline TiO_2_ were absent. And there is only a broad band around 21.5°, which is attributed to the amorphous form of TiO_2_ (Fig. 3b). Diffraction peaks of Au crystals can be clearly seen at 38.27°, 44.48° and 64.72°. Moreover, the elemental profiles of the AuNBP@TiO_2_ nanoboxes obtained from EDS are shown in Table 1. Except Ti, O, Au, and Ag elements in the composite nanostructure, other elements were from the carbon-covered Cu mesh. Therefore, it can be confirmed that the coated shell was amorphous TiO_2_.

**Table 1:** The proportion of elements in AuNBP@TiO_2_ nanoboxes

Element	Weight (%)	Element	Weight (%)
C	6.17	Fe	1.91
O	9.37	Co	0.43
Si	0.86	Cu	32.69
Ca	0.15	Ag	3.27
Ti	8.34	Au	36.81
Total	100		

**Fig. 4:**
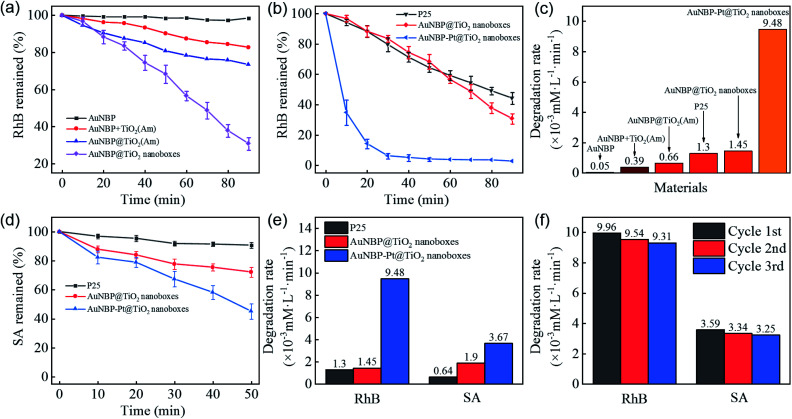
(a and b) Time course graphs and (c) degradation rate graph of the RhB degradation test of different structures, (d) time course graphs of the SA degradation test of different structures, (e) comparison chart of degradation rates, (f) the cycling test of AuNBP–Pt@TiO_2_ nanoboxes through the degradation test.

A RhB degradation experiment was used to investigate the photocatalytic capability under vis-NIR irradiation (>420 nm). The photocatalytic results are shown in Fig. 4a. Owing to poor carrier separation of pure AuNBPs, it was difficult for high-energy electrons to participate in the photocatalytic process, which resulted in poor photocatalytic activity. AuNBPs and amorphous TiO_2_ (AuNBPs + TiO_2_(Am)) mixtures show weak photocatalytic ability. The erratic contact between AuNBPs and TiO_2_(Am), and the CTAB used as a dispersant on the surface of AuNBPs affect the photocatalytic efficiency and recycling application. Comparing the AuNBP@TiO_2_ core–shell structures with the designed AuNBP@TiO_2_ nanoboxes, the nanoboxes show superior catalytic activity. Both of them were easy to uniformly disperse in water and to collect by centrifugation. The photocatalytic efficiency of the Pt-modified nanoboxes was also evaluated by the degradation of RhB (Fig. 4b). Commercial photocatalyst P25 was selected as the reference. To obtain samples with the same absorption of light, the spectra of P25 and the prepared nanobox photocatalyst were integrated in the vis-NIR region (400–1100 nm) followed by calculating the ratio between them. The samples were diluted with deionized water according to the calculation result. The particle number and mass of synthesized nanoboxes are much smaller than those of P25 at the same light absorption level because of the stronger vis-NIR light absorption of nanoboxes. Compared with other nanostructures mentioned before, the Pt-modified nanoboxes show the most excellent photocatalytic performance which was enhanced ∼6.5 times more than that of AuNBP@TiO_2_ nanoboxes, and ∼7.3 times more than that of P25 (Fig. 4c). A degradation experiment of SA was also carried out. The degradation rates of photocatalysts are shown in Fig. 4d and e. The degradation rate of P25 reduced obviously, and the reaction rate of AuNBP@TiO_2_ nanoboxes showed little change because of the different degradation process. The main difference between RhB and SA degradation is that RhB absorbs light in the vis-NIR region. Under light irradiation, electrons were excited from RhB molecules into the conduction band of TiO_2_. P25 shows photocatalytic degradation ability, even if its absorption of vis-NIR light is weak. In order to eliminate this effect, SA with no absorption in the vis-NIR light region was selected. It can be seen from the results that the catalytic efficiency of AuNBP–Pt@TiO_2_ was also affected in the case of SA. It was previously measured that amorphous TiO_2_ showed little degradation of RhB, so the additional degradation of RhB was caused by the Pt cluster modification on the surface of AuNBPs. The cycling tests of AuNBP–Pt@TiO_2_ nanoboxes (Fig. 4f) show durable activity in the photocatalysis reaction, which verified the recyclability and stability of the synthesized photocatalysts.

**Fig. 5:**
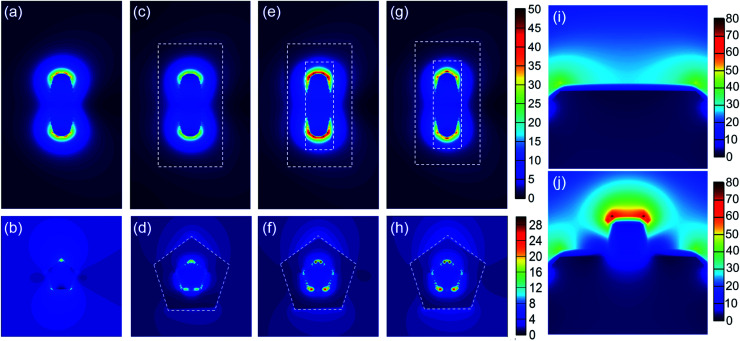
FDTD simulated electric field enhancement distribution for (a and b) AuNBPs, (c and d) AuNBP@TiO_2_ core–shell structure, (e and f) AuNBP@TiO_2_ nanoboxes, and (g and h) AuNBP–Pt@TiO_2_ nanoboxes. (a), (c), (e) and (g) show the longitudinal section electric field enhancement distribution and (b), (d), (f), and (h) show the transverse one. (i and j) Partial views of the end of AuNBPs, respectively, in AuNBP@TiO_2_ nanoboxes and AuNBP–Pt@TiO_2_ nanoboxes.

To understand the enhanced electric field caused by the SPR excitation, three-dimensional FDTD simulation was performed to calculate the spatial distributions of the electric field enhancement of the AuNBPs, AuNBP@TiO_2_ core–shell structure and AuNBP@TiO_2_ nanoboxes. As shown in Fig. 5a, the two ends of AuNBPs exhibit strong plasmon-induced electric fields caused by SPR_L_, with the enhancement of SPR_T_ as shown in Fig. 5b. After being coated with the TiO_2_(Am) shell, the nano-structure without cavities shows stronger scattering and a lower electric field than that with cavities (Fig. 5c). Fig. 5d and f confirm the enhanced electric field at the interface between AuNBPs and TiO_2_. Such nano-cavities have an enhancement effect on SPR_L_ and SPR_T_, thereby promoting the photocatalytic reaction. The Pt-modification of the nanoboxes caused a significant improvement in the photocatalytic activity, which is shown in Fig. 5g and h. After the modification, the enhanced electric field was redistributed because of the Pt clusters on AuNBPs. The longitudinal partial magnification suggested that the enhanced electric field near the Pt clusters was stronger than that on the AuNBP surface (Fig. 5i and j). Such an enhanced electric field greatly promotes the HEI process and raises the local temperature for the further reaction.

**Fig. 6:**
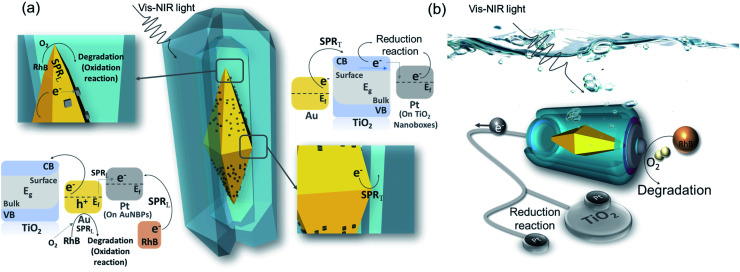
(a) Photocatalysis schematic diagram and energy band diagram; (b) model analogy diagram of the platinum modified nanoboxes.

The AuNBP–Pt@TiO_2_ nanobox design was sketched to describe the effective injection process of the hot electrons (Fig. 6a). AuNBPs provide strong light absorption for the photocatalytic reaction. Excited by vis-NIR light of a specific wavelength, an enhanced electric field is generated by SPR_T_. The plasmon resonance decays into electron–hole pairs. Upon crossing the Schottky barrier, electrons were injected into the TiO_2_ nanoboxes.^49^ Due to the bending of the energy band of TiO_2_ in contact with the noble metal,^50^ electrons will be transported away from the contact parts of the AuNBPs and TiO_2_ nanoboxes after injection because of the Schottky barrier, promoting the rapid separation of carriers.^51^ The HEI process prompted the loss of electrons and made AuNBPs positively charged which was more suitable for the oxidation reaction. Most of the electrons that were transported to TiO_2_ nanoboxes reduced Ti(iv) to Ti(iii),^52^ and some were injected into Pt clusters (located at TiO_2_ nanoboxes)^42^ which were quickly consumed by oxygen and oxidizing free-radicals. Besides rapidly absorbing electrons, the TiO_2_ nanoboxes with cavities can enhance the local electric field, which was proved by FDTD simulation. Due to the structural disorder, amorphous TiO_2_ has local electronic states in the band gap, and the optical transition of carriers between these states causes a strong increase in light absorption.^53^ Different from the AuNBP@TiO_2_ core–shell structure, the structure with partial contact facilitates the separation of charges. The cavities offered space to separate the oxidation reaction and reduction reaction. The prolonged life of photo-generated carriers promoted the progress of the photocatalytic reaction. The strong electric field from SPR_L_ increases the local temperature inside the cavities. The nanobox structure reduces the dissipation of energy.^54^ In addition, it also puts the organic molecules in a high-energy state, which increased the reaction activity of oxidation. As an excellent catalytic metal, Pt clusters on the surface of AuNBPs were higher performance active sites. A large number of dispersed Pt clusters expand the specific surface area of the photocatalytic reaction. In the metal–TiO_2_ system, positively charged noble metal atoms are more likely to attract oxygen atoms, which activates the oxygen atoms and catalyzes the oxidation reaction.^55^ A higher work function of Pt (5.65 eV) than that of Au (5.1 eV) indicates that electrons will flow from Au to Pt spontaneously, resulting in a contact potential between Pt and Au.^56,57^ In addition, the loss of electrons caused by the HEI process and the stimulation of SPR_L_ aggravated the positive charge accumulation of Au atoms. In these Au atoms, d-electron orbitals are significantly increased, contributing to a higher density of states near the Fermi level. Attributable to the redistributed and enhanced electric field, some of the Au atoms are highly oxidized by oxygen to generate Au–O bonds. The Au–O bonds in a state of high chemical energy not only participate in the oxidation reaction, but also activate the oxygen molecules to strengthen the photocatalytic activity.^55^ Because of the long-term existence of this high-energy state, Au atoms are less likely to be poisoned by the absorption of carbonate radicals, which ensures the stability of the catalytic activity of the catalyst. On the other hand, the Pt clusters with increased electron density act as additional reduction sites, which can rapidly consume electrons to promote the carrier flow.^58^ Such Pt clusters compensate to a certain extent for the lack of electron-consuming ability of amorphous TiO_2_, and also provide the possibility for AuNBP–Pt@TiO_2_ nanoboxes to participate in reduction reactions such as carbon dioxide reduction and hydrogen production.^59^ As shown in Fig. 6b, AuNBP–Pt@TiO_2_ behaves as a miniature solar electrolytic cell reaction system. AuNBPs can be regarded as batteries that supply solar energy absorption for driving the directional flow of electrons in the system. The reaction environment constructed by the TiO_2_ nanoboxes reduces the energy loss of reaction which provides high local temperature and enhanced electric field. The positive synergy of the catalyst and the co-catalyst strategically adjusted the energy transfer in the photocatalytic reaction, and significantly improved the light utilization rate and photocatalytic activity.

## Conclusion

In summary, in order to modulate the energy transfer in the photocatalytic process, we designed and fabricated a new Au–Pt@TiO_2_ nanostructure, in which AuNBPs decorated with Pt clusters were enveloped in TiO_2_ nanoboxes. The photocatalytic performance of the obtained nanostructure was remarkably improved in the degradation of RhB and SA. Based on the experimental and simulated results, the photocatalytic reaction of the photovoltaic-electrolysis model was proposed. In this nanocomposite, AuNBPs exhibit strong SPR absorption in the vis-NIR region. The HEI effect and enhanced electric field localized in the nanocavities of TiO_2_ nanoboxes promote charge separation and the hot electron injection process. The nanobox structure can effectively manage the usage of localized energy. Pt nanoclusters on the surface of AuNBPs were located in an enhanced field and promoted to a higher-level state that was appropriate for accepting and consuming electrons. Under the cooperative effects of plasmonic Au metal, catalytic Pt nanoclusters and TiO_2_ nanocavities, the photocatalytic performance of the obtained nanostructure was remarkably improved in the degradation of RhB and SA. This nanostructure not only realizes the stability of the structure, but also provides a new strategy for making full use of cooperative effects in photocatalytic performance, which offers better means for using solar energy to carry out environmental governance and develop clean energy. It also offers design of artificial photosynthesis systems with promising application prospects.

## Conflicts of interest

The authors declare no conflicts of interest.

## Supplementary Material
